# μ-Aqua-bis­(μ-4-methyl­benzoato-κ^2^ *O*:*O*′)bis­[(4-methyl­benzoato-κ*O*)(1,10-phenanthroline-κ^2^ *N*,*N*′)iron(II)]

**DOI:** 10.1107/S1600536808014207

**Published:** 2008-05-17

**Authors:** Sun Feng

**Affiliations:** a School of Chemistry and Environment, South China Normal University, Guangzhou 510006, People’s Republic of China

## Abstract

In the title binuclear complex, [Fe_2_(C_8_H_7_O_2_)_4_(C_12_H_8_N_2_)_2_(H_2_O)], the Fe^II^ ion is six-coordinated by three carboxylate O atoms from three 4-methyl­benzoate ligands, two N atoms from two 1,10-phenanthroline ligands and one bridging aqua ligand in a distorted octa­hedral geometry. The coordinated water mol­ecule acting as the bridging ligand is located on a twofold axes and the complex mol­ecule displays *C*_2_ mol­ecular symmetry. The Fe⋯Fe separation in the binuclear complex is 3.490 (3) Å. The crystal structure is stabilized by hydrogen bonding and π–π stacking inter­actions [the centroid–centroid distance between adjacent 1,10-phenanthroline ring systems is 3.653 (2) Å, and that between the 1,10-phenanthroline ring system and the phenyl ring of the 4-methyl­benzoate unit of a neighbouring complex is 3.622 (3) Å].

## Related literature

For related literature, see: Song *et al.* (2007 ▶).
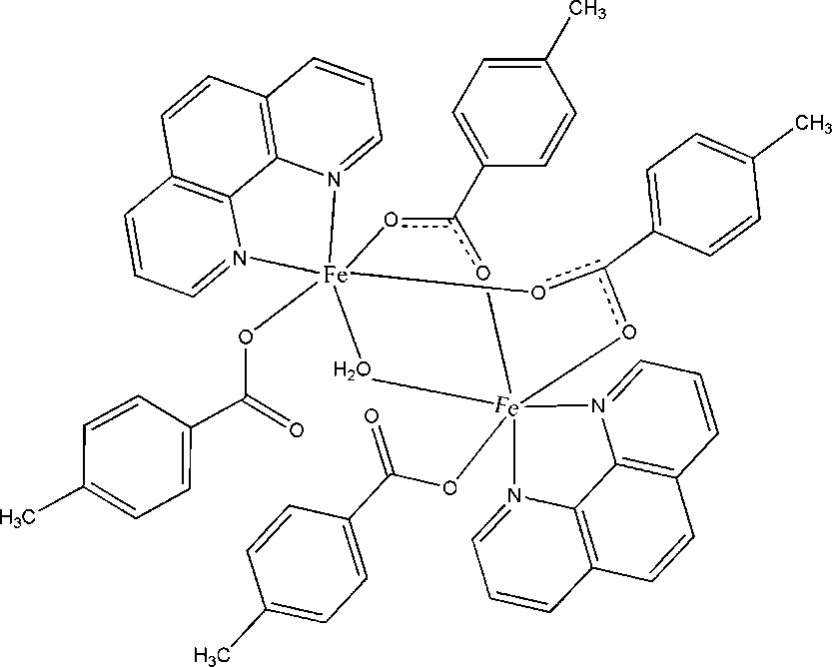


## Experimental

### 

#### Crystal data

[Fe_2_(C_8_H_7_O_2_)_4_(C_12_H_8_N_2_)_2_(H_2_O)]
*M* *_r_* = 1030.67
Monoclinic, 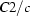

*a* = 23.1987 (6) Å
*b* = 15.7222 (4) Å
*c* = 15.6464 (4) Å
β = 121.017 (1)°
*V* = 4890.8 (2) Å^3^
*Z* = 4
Mo *K*α radiation
μ = 0.66 mm^−1^
*T* = 296 (2) K
0.20 × 0.19 × 0.16 mm

#### Data collection

Bruker APEXII area-detector diffractometer
Absorption correction: multi-scan (*SADABS*; Sheldrick, 1996 ▶) *T*_min_ = 0.880, *T*_max_ = 0.902
31900 measured reflections
5066 independent reflections
3725 reflections with *I* > 2σ(*I*)
*R*_int_ = 0.053

#### Refinement

*R*[*F* ^2^ > 2σ(*F* ^2^)] = 0.044
*wR*(*F* ^2^) = 0.133
*S* = 1.05
5066 reflections
326 parameters
2 restraints
H atoms treated by a mixture of independent and constrained refinement
Δρ_max_ = 0.30 e Å^−3^
Δρ_min_ = −0.42 e Å^−3^

### 

Data collection: *APEX2* (Bruker, 2004 ▶); cell refinement: *SAINT* (Bruker, 2004 ▶); data reduction: *SAINT*; program(s) used to solve structure: *SHELXS97* (Sheldrick, 2008 ▶); program(s) used to refine structure: *SHELXL97* (Sheldrick, 2008 ▶); molecular graphics: *XP* in *SHELXTL* (Sheldrick, 2008 ▶); software used to prepare material for publication: *SHELXTL*.

## Supplementary Material

Crystal structure: contains datablocks I, global. DOI: 10.1107/S1600536808014207/kp2167sup1.cif

Structure factors: contains datablocks I. DOI: 10.1107/S1600536808014207/kp2167Isup2.hkl

Additional supplementary materials:  crystallographic information; 3D view; checkCIF report

## Figures and Tables

**Table:** 

Fe1—O3	2.1365 (18)
Fe1—O2^i^	2.1369 (18)
Fe1—O1	2.1657 (18)
Fe1—N1	2.275 (2)
Fe1—O1*W*	2.2970 (17)
Fe1—N2	2.298 (2)

**Table:** 

O3—Fe1—O2^i^	93.91 (7)
O3—Fe1—O1	172.16 (8)
O2^i^—Fe1—O1	89.79 (7)
O3—Fe1—N1	100.73 (8)
O2^i^—Fe1—N1	86.60 (8)
O1—Fe1—N1	86.37 (7)
O3—Fe1—O1*W*	88.54 (6)
O2^i^—Fe1—O1*W*	108.77 (7)
O1—Fe1—O1*W*	83.73 (6)
N1—Fe1—O1*W*	161.62 (6)
O3—Fe1—N2	89.48 (8)
O2^i^—Fe1—N2	159.38 (8)
O1—Fe1—N2	89.47 (7)
N1—Fe1—N2	72.79 (8)
O1*W*—Fe1—N2	91.64 (7)

Symmetry code: (i) 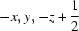.

**Table 2:** Hydrogen-bond geometry (Å, °)

*D*—H⋯*A*	*D*—H	H⋯*A*	*D*⋯*A*	*D*—H⋯*A*
O1*W*—H1*W*⋯O4^i^	0.938 (8)	1.80 (2)	2.578 (2)	138 (2)

Symmetry code: (i) 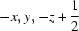.

**Fig. 1.**
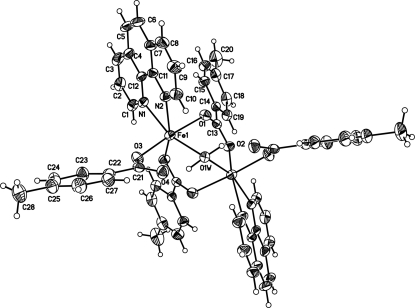
The structure of (I) showing the atomic numbering scheme. Non-H atoms are shown with the 30% probability displacement ellipsoids. Unlabelled atoms are related to the labelled atoms by the symmetry code (-x, y, 1/2 - z).

**Fig. 2.**
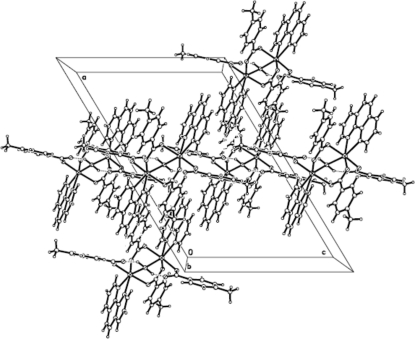
A packing view of the title compound.

## supplementary crystallographic information

### Comment

In the structural investigation of 4-methylbenzoate complexes, it has been found that the 4-methylbenzoic acid functions as a multidentate ligand [Song *et al.* (2007)] with versatile binding and coordination modes. In this report, an iron(II) complex, (I) (Fig. 1) was obtained by the reaction of 4-methylbenzoic acid, 1,10-phenanthroline and ferrous chloride in alkaline aqueous solution.

A half of the binuclear complex is an asymmetric unit where Fe^II^ ion is in the distorted octahedral geometry with the six coordinating atoms: three carboxyl O atoms from two µ_2_-bridging 4-methylbenzoate ligands and one 4-methylbenzoate ligand, two N atoms from one chelating 1,10-phenanthroline ligands, and one µ_2_-bridging aqua ligand. The Fe···Fe separation is 3.490 (3) Å. The crystal packing is *via* O—H···O hydrogen bond (Table 1) and *via* two π-π stacking interactions (Fig. 2). The centroid to centroid distance between adjacent 1,10-phenanthroline rings (*x*, –y,-1/2 + *z*) is 3.653 (2) Å, whereas the centroid to centroid distance between 1,10-phenanthroline ring and phenyl ring of 4-methylbenzoate of neighbouring complexes (1/2 - *x*, 1/2 - *y*, 1 - *z*) is 3.622 (3) Å.

### Experimental

A mixture of ferrous chloride (1 mmol), 4-methylbenzate (1 mmol), 1,10-phenanthroline (1 mmol), NaOH (1.5 mmol) and H_2_O (12 mL) was placed in a 23 mL Teflon reactor, which was heated to 433 K for three days and then cooled to room temperature at a rate of 10 K h^-1^. The crystals obtained were washed with water and dryed in air.

### Refinement

Carbon-bound H atoms were placed at calculated positions and were treated as riding on the parent C atoms with C—H = 0.93–0.97 Å, and with *U*_iso_(H) = 1.2 *U*_eq_(C). Water H atoms were tentatively located in difference Fourier maps and were refined with distance restraints of O–H = 0.82 Å, and with *U*_iso_(H) = 1.5 *U*_eq_(O).

### Crystal data

**Table:**

[Fe_2_(C_8_H_7_O_2_)_4_(C_12_H_8_N_2_)_2_(H_2_O)]	*F*_000_ = 2136
*M**_r_* = 1030.67	*D*_x_ = 1.400 Mg m^−^^3^
Monoclinic, *C*2/*c*	Mo *K*α radiation λ = 0.71073 Å
Hall symbol: -C 2yc	Cell parameters from 5300 reflections
*a* = 23.1987 (6) Å	θ = 1.3–28.0º
*b* = 15.7222 (4) Å	µ = 0.66 mm^−^^1^
*c* = 15.6464 (4) Å	*T* = 296 (2) K
β = 121.0170 (10)º	Block, colourless
*V* = 4890.8 (2) Å^3^	0.20 × 0.19 × 0.16 mm
*Z* = 4	

### Data collection

**Table:**

Bruker APEXII area-detector diffractometer	5066 independent reflections
Radiation source: fine-focus sealed tube	3725 reflections with *I* > 2σ(*I*)
Monochromator: graphite	*R*_int_ = 0.053
*T* = 296(2) K	θ_max_ = 26.5º
φ and ω scans	θ_min_ = 1.7º
Absorption correction: multi-scan(SADABS; Sheldrick, 1996)	*h* = −29→29
*T*_min_ = 0.880, *T*_max_ = 0.902	*k* = −19→19
31900 measured reflections	*l* = −19→19

### Refinement

**Table:**

Refinement on *F*^2^	Secondary atom site location: difference Fourier map
Least-squares matrix: full	Hydrogen site location: inferred from neighbouring sites
*R*[*F*^2^ > 2σ(*F*^2^)] = 0.044	H atoms treated by a mixture of independent and constrained refinement
*wR*(*F*^2^) = 0.133	*w* = 1/[σ^2^(*F*_o_^2^) + (0.0679*P*)^2^ + 3.2244*P*] where *P* = (*F*_o_^2^ + 2*F*_c_^2^)/3
*S* = 1.06	(Δ/σ)_max_ = 0.002
5066 reflections	Δρ_max_ = 0.30 e Å^−^^3^
326 parameters	Δρ_min_ = −0.42 e Å^−^^3^
2 restraints	Extinction correction: none
Primary atom site location: structure-invariant direct methods	

### Special details

**Table:**

Geometry. All e.s.d.'s (except the e.s.d. in the dihedral angle between two l.s. planes) are estimated using the full covariance matrix. The cell e.s.d.'s are taken into account individually in the estimation of e.s.d.'s in distances, angles and torsion angles; correlations between e.s.d.'s in cell parameters are only used when they are defined by crystal symmetry. An approximate (isotropic) treatment of cell e.s.d.'s is used for estimating e.s.d.'s involving l.s. planes.
Refinement. Refinement of *F*^2^ against ALL reflections. The weighted *R*-factor *wR* and goodness of fit *S* are based on *F*^2^, conventional *R*-factors *R* are based on *F*, with *F* set to zero for negative *F*^2^. The threshold expression of *F*^2^ > σ(*F*^2^) is used only for calculating *R*-factors(gt) *etc*. and is not relevant to the choice of reflections for refinement. *R*-factors based on *F*^2^ are statistically about twice as large as those based on *F*, and *R*- factors based on ALL data will be even larger.

### Fractional atomic coordinates and isotropic or equivalent isotropic displacement parameters (Å^2^)

**Table:**

	*x*	*y*	*z*	*U*_iso_*/*U*_eq_	
Fe1	0.041139 (18)	0.35596 (2)	0.37994 (3)	0.03908 (15)	
C1	0.10393 (16)	0.51103 (18)	0.5379 (2)	0.0495 (7)	
H1	0.0624	0.5365	0.4966	0.059*	
C2	0.15332 (18)	0.5574 (2)	0.6182 (2)	0.0612 (9)	
H2	0.1447	0.6126	0.6302	0.073*	
C3	0.21397 (18)	0.5211 (2)	0.6784 (2)	0.0664 (10)	
H3	0.2474	0.5515	0.7321	0.080*	
C4	0.22666 (15)	0.4382 (2)	0.6603 (2)	0.0545 (8)	
C5	0.28939 (16)	0.3946 (3)	0.7205 (2)	0.0717 (11)	
H5	0.3250	0.4229	0.7738	0.086*	
C6	0.29761 (16)	0.3135 (3)	0.7014 (2)	0.0691 (10)	
H6	0.3389	0.2871	0.7417	0.083*	
C7	0.24490 (14)	0.2671 (2)	0.6211 (2)	0.0543 (8)	
C8	0.25042 (16)	0.1818 (2)	0.5991 (3)	0.0636 (9)	
H8	0.2905	0.1524	0.6380	0.076*	
C9	0.19760 (17)	0.1427 (2)	0.5217 (3)	0.0624 (9)	
H9	0.2003	0.0855	0.5086	0.075*	
C10	0.13890 (15)	0.18871 (19)	0.4614 (2)	0.0523 (7)	
H10	0.1034	0.1616	0.4068	0.063*	
C11	0.18322 (13)	0.30873 (18)	0.55822 (19)	0.0432 (6)	
C12	0.17422 (13)	0.39593 (18)	0.57830 (19)	0.0414 (6)	
C13	0.07480 (12)	0.46759 (16)	0.25133 (19)	0.0360 (6)	
C14	0.11705 (13)	0.54627 (16)	0.28046 (19)	0.0384 (6)	
C15	0.17772 (14)	0.54973 (19)	0.3703 (2)	0.0507 (7)	
H15	0.1911	0.5041	0.4143	0.061*	
C16	0.21825 (16)	0.6204 (2)	0.3949 (3)	0.0636 (9)	
H16	0.2594	0.6209	0.4545	0.076*	
C17	0.19931 (19)	0.6901 (2)	0.3334 (3)	0.0633 (9)	
C18	0.13827 (19)	0.68755 (19)	0.2446 (3)	0.0609 (9)	
H18	0.1245	0.7344	0.2023	0.073*	
C19	0.09739 (15)	0.61659 (17)	0.2176 (2)	0.0462 (6)	
H19	0.0567	0.6158	0.1572	0.055*	
C20	0.2436 (2)	0.7685 (3)	0.3605 (4)	0.1012 (16)	
H20A	0.2891	0.7538	0.4097	0.152*	
H20B	0.2276	0.8114	0.3869	0.152*	
H20C	0.2422	0.7900	0.3020	0.152*	
C21	−0.03260 (13)	0.21381 (18)	0.4188 (2)	0.0419 (6)	
C22	−0.04954 (13)	0.17394 (19)	0.4901 (2)	0.0441 (6)	
C23	−0.05669 (16)	0.2209 (2)	0.5580 (2)	0.0582 (8)	
H23	−0.0504	0.2795	0.5608	0.070*	
C24	−0.07324 (18)	0.1821 (3)	0.6230 (3)	0.0715 (10)	
H24	−0.0791	0.2152	0.6672	0.086*	
C25	−0.08085 (18)	0.0948 (3)	0.6218 (3)	0.0680 (9)	
C26	−0.07224 (18)	0.0482 (2)	0.5554 (3)	0.0677 (9)	
H26	−0.0761	−0.0107	0.5550	0.081*	
C27	−0.05788 (15)	0.08647 (19)	0.4887 (2)	0.0545 (8)	
H27	−0.0538	0.0534	0.4428	0.065*	
C28	−0.0974 (2)	0.0524 (3)	0.6938 (3)	0.1022 (15)	
H28A	−0.0868	0.0904	0.7480	0.153*	
H28B	−0.0714	0.0012	0.7194	0.153*	
H28C	−0.1444	0.0387	0.6596	0.153*	
N1	0.11349 (11)	0.43230 (14)	0.51775 (15)	0.0415 (5)	
N2	0.13141 (10)	0.26953 (14)	0.47845 (16)	0.0411 (5)	
O1	0.09509 (9)	0.40711 (12)	0.31245 (14)	0.0455 (5)	
O2	0.02203 (9)	0.46592 (11)	0.16733 (14)	0.0449 (5)	
O3	−0.01572 (10)	0.29140 (12)	0.43251 (15)	0.0517 (5)	
O4	−0.03673 (11)	0.16889 (14)	0.35009 (15)	0.0555 (5)	
O1W	0.0000	0.26095 (16)	0.2500	0.0398 (6)	
H1W	0.0291 (12)	0.2479 (17)	0.227 (2)	0.060*	

### Atomic displacement parameters (Å^2^)

**Table:**

	*U*^11^	*U*^22^	*U*^33^	*U*^12^	*U*^13^	*U*^23^
Fe1	0.0366 (2)	0.0383 (2)	0.0366 (2)	0.00068 (15)	0.01482 (18)	0.00227 (16)
C1	0.0582 (18)	0.0447 (16)	0.0424 (15)	−0.0101 (14)	0.0236 (14)	−0.0025 (13)
C2	0.082 (2)	0.0510 (19)	0.0527 (18)	−0.0247 (17)	0.0359 (19)	−0.0115 (15)
C3	0.068 (2)	0.077 (2)	0.0430 (17)	−0.0399 (19)	0.0213 (17)	−0.0120 (17)
C4	0.0486 (17)	0.071 (2)	0.0358 (15)	−0.0213 (15)	0.0159 (14)	0.0032 (14)
C5	0.0423 (18)	0.105 (3)	0.0417 (18)	−0.0271 (19)	0.0026 (15)	0.0093 (19)
C6	0.0330 (16)	0.102 (3)	0.0511 (19)	−0.0008 (18)	0.0061 (15)	0.023 (2)
C7	0.0357 (15)	0.076 (2)	0.0480 (17)	0.0066 (14)	0.0190 (14)	0.0223 (15)
C8	0.0464 (18)	0.080 (2)	0.066 (2)	0.0248 (17)	0.0302 (18)	0.0348 (19)
C9	0.056 (2)	0.057 (2)	0.074 (2)	0.0179 (16)	0.0329 (19)	0.0174 (17)
C10	0.0472 (17)	0.0490 (17)	0.0554 (18)	0.0086 (13)	0.0226 (15)	0.0087 (14)
C11	0.0321 (13)	0.0574 (18)	0.0361 (14)	0.0012 (12)	0.0147 (12)	0.0144 (12)
C12	0.0354 (14)	0.0525 (16)	0.0314 (13)	−0.0105 (12)	0.0137 (12)	0.0046 (12)
C13	0.0333 (13)	0.0368 (14)	0.0404 (14)	−0.0016 (11)	0.0208 (12)	−0.0015 (11)
C14	0.0386 (14)	0.0401 (14)	0.0368 (14)	−0.0034 (11)	0.0197 (12)	−0.0051 (11)
C15	0.0429 (16)	0.0550 (18)	0.0469 (16)	−0.0032 (13)	0.0180 (14)	−0.0038 (14)
C16	0.0505 (19)	0.072 (2)	0.061 (2)	−0.0193 (17)	0.0235 (16)	−0.0280 (18)
C17	0.075 (2)	0.057 (2)	0.074 (2)	−0.0326 (17)	0.050 (2)	−0.0351 (18)
C18	0.093 (3)	0.0361 (16)	0.075 (2)	−0.0073 (16)	0.058 (2)	−0.0052 (15)
C19	0.0534 (17)	0.0385 (14)	0.0468 (16)	−0.0029 (13)	0.0259 (14)	−0.0041 (12)
C20	0.131 (4)	0.081 (3)	0.124 (4)	−0.067 (3)	0.089 (3)	−0.058 (3)
C21	0.0306 (13)	0.0470 (16)	0.0458 (15)	0.0021 (11)	0.0180 (12)	0.0079 (13)
C22	0.0338 (14)	0.0513 (16)	0.0443 (15)	−0.0009 (12)	0.0181 (13)	0.0071 (13)
C23	0.065 (2)	0.0578 (19)	0.0601 (19)	−0.0069 (16)	0.0380 (17)	0.0005 (16)
C24	0.079 (2)	0.087 (3)	0.066 (2)	−0.008 (2)	0.049 (2)	−0.0042 (19)
C25	0.065 (2)	0.085 (3)	0.061 (2)	−0.0109 (19)	0.0369 (18)	0.0139 (19)
C26	0.075 (2)	0.057 (2)	0.074 (2)	−0.0110 (17)	0.041 (2)	0.0135 (18)
C27	0.0579 (19)	0.0508 (18)	0.0568 (18)	−0.0047 (14)	0.0311 (16)	0.0045 (14)
C28	0.109 (3)	0.126 (4)	0.093 (3)	−0.022 (3)	0.068 (3)	0.025 (3)
N1	0.0429 (13)	0.0426 (13)	0.0329 (11)	−0.0052 (10)	0.0152 (10)	0.0019 (10)
N2	0.0356 (12)	0.0418 (12)	0.0402 (12)	0.0033 (10)	0.0155 (10)	0.0072 (10)
O1	0.0432 (11)	0.0407 (11)	0.0543 (12)	0.0012 (8)	0.0263 (9)	0.0097 (9)
O2	0.0400 (10)	0.0427 (11)	0.0442 (11)	−0.0085 (8)	0.0162 (9)	−0.0041 (8)
O3	0.0583 (12)	0.0442 (11)	0.0649 (13)	−0.0038 (9)	0.0406 (11)	0.0047 (9)
O4	0.0634 (13)	0.0565 (12)	0.0519 (12)	−0.0118 (10)	0.0334 (11)	−0.0035 (10)
O1W	0.0425 (15)	0.0403 (14)	0.0374 (14)	0.000	0.0211 (12)	0.000

### Geometric parameters (Å, °)

**Table:**

Fe1—O3	2.1365 (18)	C14—C19	1.391 (4)
Fe1—O2^i^	2.1369 (18)	C15—C16	1.376 (4)
Fe1—O1	2.1657 (18)	C15—H15	0.9300
Fe1—N1	2.275 (2)	C16—C17	1.373 (5)
Fe1—O1W	2.2970 (17)	C16—H16	0.9300
Fe1—N2	2.298 (2)	C17—C18	1.382 (5)
C1—N1	1.324 (4)	C17—C20	1.519 (4)
C1—C2	1.393 (4)	C18—C19	1.382 (4)
C1—H1	0.9300	C18—H18	0.9300
C2—C3	1.352 (5)	C19—H19	0.9300
C2—H2	0.9300	C20—H20A	0.9600
C3—C4	1.398 (5)	C20—H20B	0.9600
C3—H3	0.9300	C20—H20C	0.9600
C4—C12	1.400 (4)	C21—O4	1.248 (3)
C4—C5	1.436 (5)	C21—O3	1.265 (3)
C5—C6	1.346 (5)	C21—C22	1.499 (4)
C5—H5	0.9300	C22—C23	1.372 (4)
C6—C7	1.422 (5)	C22—C27	1.387 (4)
C6—H6	0.9300	C23—C24	1.398 (4)
C7—C8	1.406 (5)	C23—H23	0.9300
C7—C11	1.412 (4)	C24—C25	1.383 (5)
C8—C9	1.349 (5)	C24—H24	0.9300
C8—H8	0.9300	C25—C26	1.367 (5)
C9—C10	1.395 (4)	C25—C28	1.519 (5)
C9—H9	0.9300	C26—C27	1.387 (4)
C10—N2	1.328 (4)	C26—H26	0.9300
C10—H10	0.9300	C27—H27	0.9300
C11—N2	1.355 (3)	C28—H28A	0.9600
C11—C12	1.446 (4)	C28—H28B	0.9600
C12—N1	1.354 (3)	C28—H28C	0.9600
C13—O2	1.252 (3)	O2—Fe1^i^	2.1369 (18)
C13—O1	1.256 (3)	O1W—Fe1^i^	2.2970 (17)
C13—C14	1.496 (3)	O1W—H1W	0.938 (8)
C14—C15	1.386 (4)		
			
O3—Fe1—O2^i^	93.91 (7)	C14—C15—H15	119.8
O3—Fe1—O1	172.16 (8)	C17—C16—C15	121.6 (3)
O2^i^—Fe1—O1	89.79 (7)	C17—C16—H16	119.2
O3—Fe1—N1	100.73 (8)	C15—C16—H16	119.2
O2^i^—Fe1—N1	86.60 (8)	C16—C17—C18	118.3 (3)
O1—Fe1—N1	86.37 (7)	C16—C17—C20	121.8 (4)
O3—Fe1—O1W	88.54 (6)	C18—C17—C20	120.0 (4)
O2^i^—Fe1—O1W	108.77 (7)	C19—C18—C17	121.2 (3)
O1—Fe1—O1W	83.73 (6)	C19—C18—H18	119.4
N1—Fe1—O1W	161.62 (6)	C17—C18—H18	119.4
O3—Fe1—N2	89.48 (8)	C18—C19—C14	120.1 (3)
O2^i^—Fe1—N2	159.38 (8)	C18—C19—H19	119.9
O1—Fe1—N2	89.47 (7)	C14—C19—H19	119.9
N1—Fe1—N2	72.79 (8)	C17—C20—H20A	109.5
O1W—Fe1—N2	91.64 (7)	C17—C20—H20B	109.5
N1—C1—C2	122.9 (3)	H20A—C20—H20B	109.5
N1—C1—H1	118.5	C17—C20—H20C	109.5
C2—C1—H1	118.5	H20A—C20—H20C	109.5
C3—C2—C1	119.0 (3)	H20B—C20—H20C	109.5
C3—C2—H2	120.5	O4—C21—O3	124.9 (3)
C1—C2—H2	120.5	O4—C21—C22	118.1 (3)
C2—C3—C4	120.3 (3)	O3—C21—C22	117.0 (3)
C2—C3—H3	119.8	C23—C22—C27	118.4 (3)
C4—C3—H3	119.8	C23—C22—C21	122.3 (3)
C3—C4—C12	117.0 (3)	C27—C22—C21	119.4 (3)
C3—C4—C5	124.1 (3)	C22—C23—C24	121.0 (3)
C12—C4—C5	118.9 (3)	C22—C23—H23	119.5
C6—C5—C4	121.2 (3)	C24—C23—H23	119.5
C6—C5—H5	119.4	C25—C24—C23	120.3 (3)
C4—C5—H5	119.4	C25—C24—H24	119.8
C5—C6—C7	121.8 (3)	C23—C24—H24	119.8
C5—C6—H6	119.1	C26—C25—C24	118.3 (3)
C7—C6—H6	119.1	C26—C25—C28	121.3 (4)
C8—C7—C11	117.3 (3)	C24—C25—C28	120.4 (4)
C8—C7—C6	124.0 (3)	C25—C26—C27	121.7 (3)
C11—C7—C6	118.6 (3)	C25—C26—H26	119.2
C9—C8—C7	120.0 (3)	C27—C26—H26	119.2
C9—C8—H8	120.0	C26—C27—C22	120.2 (3)
C7—C8—H8	120.0	C26—C27—H27	119.9
C8—C9—C10	119.3 (3)	C22—C27—H27	119.9
C8—C9—H9	120.4	C25—C28—H28A	109.5
C10—C9—H9	120.4	C25—C28—H28B	109.5
N2—C10—C9	123.0 (3)	H28A—C28—H28B	109.5
N2—C10—H10	118.5	C25—C28—H28C	109.5
C9—C10—H10	118.5	H28A—C28—H28C	109.5
N2—C11—C7	122.0 (3)	H28B—C28—H28C	109.5
N2—C11—C12	118.2 (2)	C1—N1—C12	118.0 (2)
C7—C11—C12	119.8 (3)	C1—N1—Fe1	125.91 (19)
N1—C12—C4	122.7 (3)	C12—N1—Fe1	115.70 (18)
N1—C12—C11	117.6 (2)	C10—N2—C11	118.3 (2)
C4—C12—C11	119.6 (3)	C10—N2—Fe1	126.82 (19)
O2—C13—O1	124.3 (2)	C11—N2—Fe1	114.71 (17)
O2—C13—C14	118.0 (2)	C13—O1—Fe1	124.66 (16)
O1—C13—C14	117.7 (2)	C13—O2—Fe1^i^	120.36 (16)
C15—C14—C19	118.5 (3)	C21—O3—Fe1	126.24 (18)
C15—C14—C13	120.3 (2)	Fe1—O1W—Fe1^i^	98.87 (10)
C19—C14—C13	121.1 (2)	Fe1—O1W—H1W	115.5 (18)
C16—C15—C14	120.3 (3)	Fe1^i^—O1W—H1W	81.6 (18)
C16—C15—H15	119.8		

Symmetry codes: (i) −*x*, *y*, −*z*+1/2.

### Hydrogen-bond geometry (Å, °)

**Table:**

*D*—H···*A*	*D*—H	H···*A*	*D*···*A*	*D*—H···*A*
O1W—H1W···O4^i^	0.938 (8)	1.80 (2)	2.578 (2)	138 (2)

Symmetry codes: (i) −*x*, *y*, −*z*+1/2.
